# Tetra­aqua­(5,5′-dimethyl-2,2′-bipyridine-κ^2^ *N*,*N*′)zinc(II) sulfate

**DOI:** 10.1107/S160053680902488X

**Published:** 2009-07-04

**Authors:** Qing-Lan Zhao, Hui-Feng Bai

**Affiliations:** a College of Chemistry and Chemical Engineering, Henan University, Kaifeng 475001, Henan, People’s Republic of China; b Institute of Molecular and Crystal Engineering, College of Chemistry and Chemical Engineering, Henan University, Kaifeng 475001, Henan, People’s Republic of China

## Abstract

The asymmetric unit of the title compound, [Zn(C_12_H_12_N_2_)(H_2_O)_4_]SO_4_, consists of a Zn^II^ complex cation, a sulfate anion and four mol­ecules of water coordinated to the Zn^II^ atom. The Zn^II^ complex cation, with approximate twofold symmetry, displays a slightly distorted octa­hedral geometry around the Zn^II^ atom, which is coordinated by two N atoms from a 5,5′-dimethyl-2,2′-bipyridine ligand and by the O atoms of four water mol­ecules. In the crystal, O—H⋯O hydrogen bonds help to establish the packing.

## Related literature

For related structures, see: Schubert, Eschbaumer *et al.* (1999 ▶); Schubert, Hochwimmer *et al.* (1999 ▶); Shi *et al.* (2009 ▶); Zhang *et al.* (2009 ▶); Momeni *et al.* (2009 ▶); Kim *et al.* (2009 ▶); Yang *et al.* (2001 ▶).
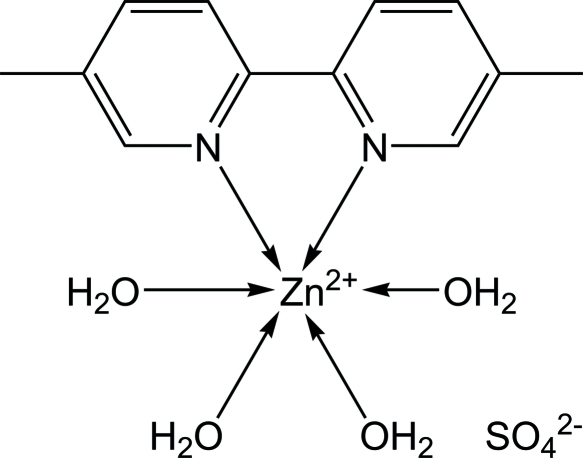


## Experimental

### 

#### Crystal data

[Zn(C_12_H_12_N_2_)(H_2_O)_4_]SO_4_
*M* *_r_* = 417.73
Monoclinic, 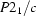

*a* = 9.5648 (17) Å
*b* = 9.6050 (17) Å
*c* = 18.477 (3) Å
β = 102.453 (4)°
*V* = 1657.5 (5) Å^3^
*Z* = 4
Mo *K*α radiation
μ = 1.65 mm^−1^
*T* = 296 K
0.30 × 0.26 × 0.25 mm

#### Data collection

Bruker SMART APEXII CCD area-detector diffractometer
Absorption correction: multi-scan (*SADABS*; Bruker, 2005 ▶) *T*_min_ = 0.637, *T*_max_ = 0.683
9462 measured reflections
3263 independent reflections
2648 reflections with *I* > 2σ(*I*)
*R*_int_ = 0.085

#### Refinement

*R*[*F* ^2^ > 2σ(*F* ^2^)] = 0.037
*wR*(*F* ^2^) = 0.091
*S* = 1.04
3263 reflections
219 parameters
H-atom parameters constrained
Δρ_max_ = 0.52 e Å^−3^
Δρ_min_ = −0.40 e Å^−3^

### 

Data collection: *APEX2* (Bruker, 2005 ▶); cell refinement: *SAINT* (Bruker, 2005 ▶); data reduction: *SAINT*; program(s) used to solve structure: *SHELXS97* (Sheldrick, 2008 ▶); program(s) used to refine structure: *SHELXL97* (Sheldrick, 2008 ▶); molecular graphics: *SHELXTL* (Sheldrick, 2008 ▶); software used to prepare material for publication: *SHELXTL*.

## Supplementary Material

Crystal structure: contains datablocks I, global. DOI: 10.1107/S160053680902488X/pv2173sup1.cif

Structure factors: contains datablocks I. DOI: 10.1107/S160053680902488X/pv2173Isup2.hkl

Additional supplementary materials:  crystallographic information; 3D view; checkCIF report

## Figures and Tables

**Table 1:** Hydrogen-bond geometry (Å, °)

*D*—H⋯*A*	*D*—H	H⋯*A*	*D*⋯*A*	*D*—H⋯*A*
O4*W*—H4*WB*⋯O6^i^	0.85	1.84	2.695 (3)	179
O4*W*—H4*WA*⋯O5	0.85	1.87	2.722 (3)	178
O3*W*—H3*WB*⋯O5^ii^	0.85	1.90	2.748 (3)	178
O3*W*—H3*WA*⋯O6	0.85	1.96	2.804 (3)	170
O2*W*—H2*WB*⋯O8^i^	0.85	1.99	2.831 (3)	170
O2*W*—H2*WA*⋯O7^iii^	0.85	1.92	2.766 (3)	173
O1*W*—H1*WB*⋯O8^iii^	0.85	1.87	2.717 (3)	175
O1*W*—H1*WA*⋯O7^ii^	0.85	1.93	2.772 (3)	169

Symmetry codes: (i) 
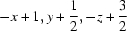
; (ii) 
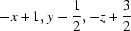
; (iii) 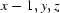.

**Fig. 1.**
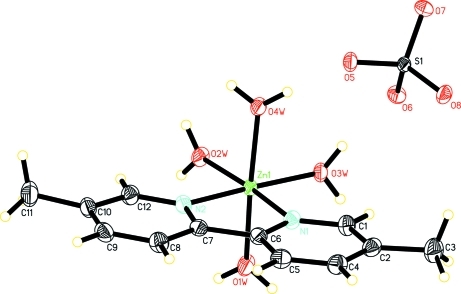
The molecular structure of the title compound with the atom-labelling scheme. Displacement ellipsoids are drawn at the 30% probability level. H atoms are presented as small spheres of arbitrary radius.

**Fig. 2.**
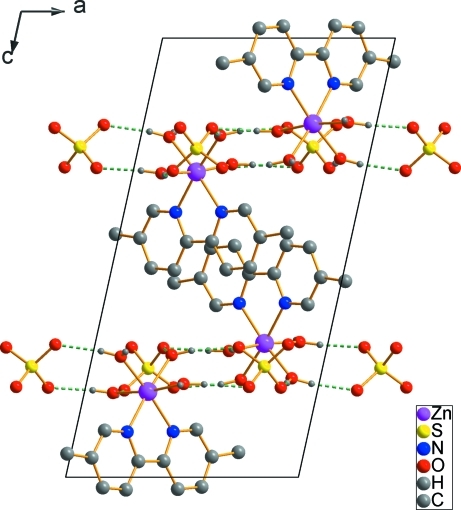
Crystal packing of the title complound. Hydrogen-bond interactions are drawn with dashed lines.

## supplementary crystallographic information

### Comment

As a contribution to structural characterization of 5,5'-dimethyl-2,2'-bipyridine complexes (Schubert, Eschbaumer *et al.* 1999; Schubert, Hochwimmer *et al.* 1999; Yang *et al.*,  2001) we present here the crystal structure of the title complex, [ZnL(H_2_O)_4_].SO_4_ (*L* = 5,5'-dimethyl-2,2'-bipyridine).

The molecular structure of the title compound (Fig. 1) is made up of a [ZnL(H_2_O)_4_]^2+^ cation and a sulfate anion; the cation shows an approximate two fold rotational symmetry. The Zinc atom is coordinated totwo N atoms of a 5,5'-dimethyl-2,2'-bipyridine ligand and four aqua ligands to form distorted octahedral geometry.

With O—H···O and O—H···S hydrogen bonds (Table 1), a three-dimensional network is formed as shown in Fig. 2.

### Experimental

The title compound was synthesized hydrothermally in a Teflon-lined autoclave (25 ml) by heating a mixture of 5,5'-dimethyl-2,2'-bipyridine (0.2 mmol), ZnSO_4_ (0.2 mmol) and one drop of Et_3_N (pH ≈ 8–9) in water (10 ml) at 393 K for 3 d. Crystals suitable for X-ray analysis were obtained.

### Refinement

All H atoms were included in calculated positions, with C—H bond lengths fixed at 0.96 Å (methyl CH_3_), 0.93Å (aryl group) and O—H = 0.85 Å and were refined in the riding-model approximation. *U*_iso_(H) values were calculated at 1.5 *U*_eq_(C) for methyl groups and 1.2 *U*_eq_(C) otherwise.

### Crystal data

**Table:**

[Zn(C_12_H_12_N_2_)(H_2_O)_4_]SO_4_	*F*(000) = 864
*M**_r_* = 417.73	*D*_x_ = 1.674 Mg m^−^^3^
Monoclinic, *P*2_1_/*c*	Mo *K*α radiation, λ = 0.71073 Å
Hall symbol: -P 2ybc	Cell parameters from 4417 reflections
*a* = 9.5648 (17) Å	θ = 2.2–27.9°
*b* = 9.6050 (17) Å	µ = 1.65 mm^−^^1^
*c* = 18.477 (3) Å	*T* = 296 K
β = 102.453 (4)°	Block, colourless
*V* = 1657.5 (5) Å^3^	0.30 × 0.26 × 0.25 mm
*Z* = 4	

### Data collection

**Table:**

Bruker SMART APEXII CCD area-detector diffractometer	3263 independent reflections
Radiation source: fine-focus sealed tube	2648 reflections with *I* > 2σ(*I*)
graphite	*R*_int_ = 0.085
φ and ω scans	θ_max_ = 26.0°, θ_min_ = 2.2°
Absorption correction: multi-scan (*SADABS*; Bruker, 2005)	*h* = −11→11
*T*_min_ = 0.637, *T*_max_ = 0.683	*k* = −11→10
9462 measured reflections	*l* = −22→13

### Refinement

**Table:**

Refinement on *F*^2^	Primary atom site location: structure-invariant direct methods
Least-squares matrix: full	Secondary atom site location: difference Fourier map
*R*[*F*^2^ > 2σ(*F*^2^)] = 0.037	Hydrogen site location: inferred from neighbouring sites
*wR*(*F*^2^) = 0.091	H-atom parameters constrained
*S* = 1.04	*w* = 1/[σ^2^(*F*_o_^2^) + (0.041*P*)^2^] where *P* = (*F*_o_^2^ + 2*F*_c_^2^)/3
3263 reflections	(Δ/σ)_max_ = 0.001
219 parameters	Δρ_max_ = 0.52 e Å^−^^3^
0 restraints	Δρ_min_ = −0.39 e Å^−^^3^

### Special details

**Table:**

Geometry. All e.s.d.'s (except the e.s.d. in the dihedral angle between two l.s. planes) are estimated using the full covariance matrix. The cell e.s.d.'s are taken into account individually in the estimation of e.s.d.'s in distances, angles and torsion angles; correlations between e.s.d.'s in cell parameters are only used when they are defined by crystal symmetry. An approximate (isotropic) treatment of cell e.s.d.'s is used for estimating e.s.d.'s involving l.s. planes.
Refinement. Refinement of *F*^2^ against ALL reflections. The weighted *R*-factor *wR* and goodness of fit *S* are based on *F*^2^, conventional *R*-factors *R* are based on *F*, with *F* set to zero for negative *F*^2^. The threshold expression of *F*^2^ > σ(*F*^2^) is used only for calculating *R*-factors(gt) *etc*. and is not relevant to the choice of reflections for refinement. *R*-factors based on *F*^2^ are statistically about twice as large as those based on *F*, and *R*- factors based on ALL data will be even larger.

### Fractional atomic coordinates and isotropic or equivalent isotropic displacement parameters (Å^2^)

**Table:**

	*x*	*y*	*z*	*U*_iso_*/*U*_eq_	
Zn1	0.27251 (3)	0.86942 (3)	0.804800 (16)	0.02610 (12)	
S1	0.74896 (6)	0.86596 (7)	0.74789 (4)	0.02499 (17)	
N1	0.4185 (2)	0.7784 (2)	0.89612 (12)	0.0321 (5)	
N2	0.2216 (2)	0.9768 (2)	0.89671 (12)	0.0324 (5)	
O1W	0.1234 (2)	0.7150 (2)	0.80803 (15)	0.0632 (7)	
H1WA	0.1400	0.6281	0.8125	0.076*	
H1WB	0.0336	0.7268	0.8018	0.076*	
O2W	0.12257 (18)	0.9747 (2)	0.72531 (10)	0.0378 (5)	
H2WA	0.0329	0.9606	0.7130	0.045*	
H2WB	0.1381	1.0586	0.7146	0.045*	
O3W	0.33946 (19)	0.7568 (2)	0.72175 (10)	0.0347 (5)	
H3WA	0.4203	0.7743	0.7118	0.042*	
H3WB	0.3264	0.6693	0.7182	0.042*	
O4W	0.4183 (2)	1.0182 (2)	0.78745 (13)	0.0486 (6)	
H4WA	0.5066	1.0052	0.7884	0.058*	
H4WB	0.4049	1.1053	0.7901	0.058*	
O5	0.70202 (19)	0.9743 (2)	0.79426 (10)	0.0335 (4)	
O6	0.62104 (18)	0.7951 (2)	0.70442 (10)	0.0343 (4)	
O7	0.83003 (19)	0.9308 (2)	0.69779 (11)	0.0359 (5)	
O8	0.83933 (19)	0.7631 (2)	0.79580 (10)	0.0358 (5)	
C1	0.5164 (3)	0.6810 (3)	0.89163 (17)	0.0369 (7)	
H1A	0.5233	0.6498	0.8449	0.044*	
C2	0.6085 (3)	0.6240 (3)	0.95322 (17)	0.0379 (7)	
C3	0.7185 (3)	0.5184 (4)	0.9430 (2)	0.0527 (9)	
H3A	0.6773	0.4559	0.9036	0.079*	
H3B	0.7494	0.4667	0.9881	0.079*	
H3C	0.7991	0.5652	0.9308	0.079*	
C4	0.5934 (3)	0.6713 (3)	1.02187 (17)	0.0409 (7)	
H4A	0.6511	0.6351	1.0648	0.049*	
C5	0.4932 (3)	0.7720 (3)	1.02696 (15)	0.0393 (7)	
H5	0.4843	0.8043	1.0732	0.047*	
C6	0.4054 (3)	0.8254 (3)	0.96315 (15)	0.0311 (6)	
C7	0.2972 (3)	0.9349 (3)	0.96385 (15)	0.0303 (6)	
C8	0.2708 (3)	0.9936 (3)	1.02828 (16)	0.0399 (7)	
H8	0.3214	0.9634	1.0743	0.048*	
C9	0.1693 (3)	1.0966 (3)	1.02349 (18)	0.0419 (7)	
H9	0.1517	1.1360	1.0666	0.050*	
C10	0.0931 (3)	1.1423 (3)	0.95547 (18)	0.0384 (7)	
C11	−0.0155 (4)	1.2571 (4)	0.9464 (2)	0.0544 (9)	
H11A	−0.0502	1.2665	0.9911	0.082*	
H11B	−0.0938	1.2353	0.9060	0.082*	
H11C	0.0281	1.3428	0.9362	0.082*	
C12	0.1239 (3)	1.0770 (3)	0.89353 (17)	0.0371 (7)	
H12	0.0732	1.1048	0.8470	0.045*	

### Atomic displacement parameters (Å^2^)

**Table:**

	*U*^11^	*U*^22^	*U*^33^	*U*^12^	*U*^13^	*U*^23^
Zn1	0.01825 (17)	0.02401 (18)	0.0354 (2)	0.00040 (12)	0.00430 (11)	−0.00115 (13)
S1	0.0161 (3)	0.0206 (3)	0.0387 (4)	0.0001 (2)	0.0068 (2)	−0.0004 (3)
N1	0.0287 (12)	0.0324 (12)	0.0335 (13)	0.0039 (10)	0.0029 (9)	0.0026 (11)
N2	0.0292 (12)	0.0330 (12)	0.0359 (14)	0.0003 (10)	0.0089 (10)	−0.0031 (11)
O1W	0.0231 (11)	0.0266 (12)	0.143 (2)	−0.0025 (10)	0.0252 (12)	−0.0011 (13)
O2W	0.0200 (9)	0.0340 (11)	0.0553 (13)	0.0050 (8)	−0.0006 (8)	0.0072 (10)
O3W	0.0266 (10)	0.0263 (10)	0.0534 (12)	0.0023 (8)	0.0134 (8)	−0.0075 (9)
O4W	0.0229 (10)	0.0246 (10)	0.1025 (18)	−0.0007 (9)	0.0230 (10)	0.0024 (11)
O5	0.0294 (10)	0.0277 (10)	0.0452 (12)	0.0053 (8)	0.0116 (8)	−0.0061 (9)
O6	0.0203 (9)	0.0336 (11)	0.0478 (12)	−0.0079 (8)	0.0050 (8)	−0.0051 (9)
O7	0.0282 (10)	0.0343 (11)	0.0480 (12)	−0.0060 (9)	0.0148 (8)	0.0016 (9)
O8	0.0270 (10)	0.0270 (10)	0.0522 (13)	0.0076 (8)	0.0058 (8)	0.0053 (9)
C1	0.0317 (15)	0.0394 (16)	0.0392 (17)	0.0061 (13)	0.0064 (12)	−0.0003 (14)
C2	0.0318 (15)	0.0325 (16)	0.0469 (19)	−0.0001 (13)	0.0033 (12)	0.0034 (14)
C3	0.0392 (18)	0.052 (2)	0.064 (2)	0.0148 (16)	0.0058 (14)	0.0063 (18)
C4	0.0316 (16)	0.0439 (17)	0.0438 (19)	0.0053 (14)	0.0006 (12)	0.0127 (15)
C5	0.0425 (17)	0.0427 (17)	0.0320 (17)	−0.0004 (15)	0.0062 (12)	0.0031 (14)
C6	0.0264 (14)	0.0307 (14)	0.0361 (16)	−0.0012 (12)	0.0065 (11)	0.0012 (13)
C7	0.0261 (14)	0.0304 (14)	0.0340 (16)	−0.0036 (12)	0.0057 (11)	−0.0008 (12)
C8	0.0412 (17)	0.0450 (18)	0.0337 (18)	0.0020 (15)	0.0083 (13)	−0.0008 (14)
C9	0.0440 (18)	0.0430 (17)	0.0437 (19)	−0.0030 (15)	0.0205 (14)	−0.0076 (15)
C10	0.0296 (15)	0.0363 (16)	0.0520 (19)	0.0004 (13)	0.0149 (13)	−0.0077 (14)
C11	0.049 (2)	0.049 (2)	0.068 (2)	0.0133 (17)	0.0188 (16)	−0.0089 (17)
C12	0.0306 (15)	0.0380 (16)	0.0417 (17)	0.0036 (13)	0.0053 (12)	0.0017 (14)

### Geometric parameters (Å, °)

**Table:**

Zn1—O1W	2.068 (2)	C1—H1A	0.9300
Zn1—O4W	2.0693 (19)	C2—C4	1.384 (4)
Zn1—O2W	2.0806 (18)	C2—C3	1.502 (4)
Zn1—O3W	2.0880 (17)	C3—H3A	0.9600
Zn1—N2	2.131 (2)	C3—H3B	0.9600
Zn1—N1	2.132 (2)	C3—H3C	0.9600
S1—O7	1.4682 (19)	C4—C5	1.379 (4)
S1—O8	1.4756 (19)	C4—H4A	0.9300
S1—O6	1.4772 (19)	C5—C6	1.389 (4)
S1—O5	1.4779 (19)	C5—H5	0.9300
N1—C1	1.339 (3)	C6—C7	1.477 (4)
N1—C6	1.349 (3)	C7—C8	1.388 (4)
N2—C12	1.334 (4)	C8—C9	1.376 (4)
N2—C7	1.355 (3)	C8—H8	0.9300
O1W—H1WA	0.8496	C9—C10	1.381 (4)
O1W—H1WB	0.8495	C9—H9	0.9300
O2W—H2WA	0.8497	C10—C12	1.392 (4)
O2W—H2WB	0.8497	C10—C11	1.499 (4)
O3W—H3WA	0.8497	C11—H11A	0.9600
O3W—H3WB	0.8498	C11—H11B	0.9600
O4W—H4WA	0.8498	C11—H11C	0.9600
O4W—H4WB	0.8499	C12—H12	0.9300
C1—C2	1.392 (4)		
			
O1W—Zn1—O4W	172.74 (10)	C2—C1—H1A	118.3
O1W—Zn1—O2W	89.68 (9)	C4—C2—C1	116.5 (3)
O4W—Zn1—O2W	86.56 (8)	C4—C2—C3	123.5 (3)
O1W—Zn1—O3W	88.40 (9)	C1—C2—C3	120.0 (3)
O4W—Zn1—O3W	85.42 (8)	C2—C3—H3A	109.5
O2W—Zn1—O3W	90.41 (8)	C2—C3—H3B	109.5
O1W—Zn1—N2	92.68 (9)	H3A—C3—H3B	109.5
O4W—Zn1—N2	93.82 (9)	C2—C3—H3C	109.5
O2W—Zn1—N2	94.95 (8)	H3A—C3—H3C	109.5
O3W—Zn1—N2	174.54 (8)	H3B—C3—H3C	109.5
O1W—Zn1—N1	91.22 (10)	C5—C4—C2	120.3 (3)
O4W—Zn1—N1	93.29 (9)	C5—C4—H4A	119.9
O2W—Zn1—N1	172.85 (8)	C2—C4—H4A	119.9
O3W—Zn1—N1	96.70 (8)	C4—C5—C6	120.2 (3)
N2—Zn1—N1	77.93 (9)	C4—C5—H5	119.9
O7—S1—O8	109.97 (11)	C6—C5—H5	119.9
O7—S1—O6	109.92 (12)	N1—C6—C5	119.8 (3)
O8—S1—O6	109.07 (11)	N1—C6—C7	116.8 (2)
O7—S1—O5	109.56 (11)	C5—C6—C7	123.5 (3)
O8—S1—O5	109.62 (11)	N2—C7—C8	120.3 (3)
O6—S1—O5	108.68 (11)	N2—C7—C6	116.1 (2)
C1—N1—C6	119.7 (2)	C8—C7—C6	123.6 (3)
C1—N1—Zn1	125.80 (19)	C9—C8—C7	119.5 (3)
C6—N1—Zn1	114.48 (17)	C9—C8—H8	120.2
C12—N2—C7	119.0 (2)	C7—C8—H8	120.2
C12—N2—Zn1	126.3 (2)	C8—C9—C10	120.9 (3)
C7—N2—Zn1	114.67 (18)	C8—C9—H9	119.5
Zn1—O1W—H1WA	126.3	C10—C9—H9	119.5
Zn1—O1W—H1WB	125.8	C9—C10—C12	116.2 (3)
H1WA—O1W—H1WB	107.8	C9—C10—C11	123.6 (3)
Zn1—O2W—H2WA	127.8	C12—C10—C11	120.3 (3)
Zn1—O2W—H2WB	119.8	C10—C11—H11A	109.5
H2WA—O2W—H2WB	107.8	C10—C11—H11B	109.5
Zn1—O3W—H3WA	119.6	H11A—C11—H11B	109.5
Zn1—O3W—H3WB	120.4	C10—C11—H11C	109.5
H3WA—O3W—H3WB	107.8	H11A—C11—H11C	109.5
Zn1—O4W—H4WA	126.5	H11B—C11—H11C	109.5
Zn1—O4W—H4WB	123.8	N2—C12—C10	124.0 (3)
H4WA—O4W—H4WB	107.7	N2—C12—H12	118.0
N1—C1—C2	123.5 (3)	C10—C12—H12	118.0
N1—C1—H1A	118.3		
			
O1W—Zn1—N1—C1	88.9 (2)	C1—N1—C6—C5	−0.2 (4)
O4W—Zn1—N1—C1	−85.4 (2)	Zn1—N1—C6—C5	178.9 (2)
O3W—Zn1—N1—C1	0.3 (2)	C1—N1—C6—C7	178.8 (2)
N2—Zn1—N1—C1	−178.6 (2)	Zn1—N1—C6—C7	−2.1 (3)
O1W—Zn1—N1—C6	−90.20 (19)	C4—C5—C6—N1	−0.1 (4)
O4W—Zn1—N1—C6	95.50 (19)	C4—C5—C6—C7	−178.9 (3)
O3W—Zn1—N1—C6	−178.73 (18)	C12—N2—C7—C8	1.4 (4)
N2—Zn1—N1—C6	2.29 (18)	Zn1—N2—C7—C8	−177.9 (2)
O1W—Zn1—N2—C12	−90.7 (2)	C12—N2—C7—C6	−179.0 (2)
O4W—Zn1—N2—C12	86.1 (2)	Zn1—N2—C7—C6	1.8 (3)
O2W—Zn1—N2—C12	−0.8 (2)	N1—C6—C7—N2	0.2 (4)
N1—Zn1—N2—C12	178.6 (2)	C5—C6—C7—N2	179.1 (2)
O1W—Zn1—N2—C7	88.50 (19)	N1—C6—C7—C8	179.9 (3)
O4W—Zn1—N2—C7	−94.72 (19)	C5—C6—C7—C8	−1.2 (4)
O2W—Zn1—N2—C7	178.41 (18)	N2—C7—C8—C9	−1.3 (4)
N1—Zn1—N2—C7	−2.17 (18)	C6—C7—C8—C9	179.1 (3)
C6—N1—C1—C2	−0.4 (4)	C7—C8—C9—C10	0.1 (4)
Zn1—N1—C1—C2	−179.4 (2)	C8—C9—C10—C12	0.9 (4)
N1—C1—C2—C4	1.1 (4)	C8—C9—C10—C11	−178.0 (3)
N1—C1—C2—C3	−178.0 (3)	C7—N2—C12—C10	−0.3 (4)
C1—C2—C4—C5	−1.3 (4)	Zn1—N2—C12—C10	178.9 (2)
C3—C2—C4—C5	177.8 (3)	C9—C10—C12—N2	−0.9 (4)
C2—C4—C5—C6	0.8 (4)	C11—C10—C12—N2	178.1 (3)

### Hydrogen-bond geometry (Å, °)

**Table:**

*D*—H···*A*	*D*—H	H···*A*	*D*···*A*	*D*—H···*A*
O4W—H4WB···S1^i^	0.85	2.91	3.701 (2)	155
O4W—H4WB···O6^i^	0.85	1.84	2.695 (3)	179
O4W—H4WA···S1	0.85	2.91	3.697 (2)	155
O4W—H4WA···O5	0.85	1.87	2.722 (3)	178
O3W—H3WB···O5^ii^	0.85	1.90	2.748 (3)	178
O3W—H3WA···O6	0.85	1.96	2.804 (3)	170
O2W—H2WB···O8^i^	0.85	1.99	2.831 (3)	170
O2W—H2WA···O7^iii^	0.85	1.92	2.766 (3)	173
O1W—H1WB···S1^iii^	0.85	3.00	3.801 (2)	157
O1W—H1WB···O8^iii^	0.85	1.87	2.717 (3)	175
O1W—H1WA···O7^ii^	0.85	1.93	2.772 (3)	169

Symmetry codes: (i) −*x*+1, *y*+1/2, −*z*+3/2; (ii) −*x*+1, *y*−1/2, −*z*+3/2; (iii) *x*−1, *y*, *z*.
